# Treating Initial and Recurrent *C. difficile*: A Retrospective Analysis of 100 Referred Patients

**DOI:** 10.3390/microorganisms14040911

**Published:** 2026-04-17

**Authors:** Rahim A. Burdette, Caroline C. Whitt, Krystyna J. Cios Phillips, Mark T. Worthington, Brian W. Behm, Cirle A. Warren

**Affiliations:** 1 School of Medicine, University of Virginia, Charlottesville, VA 22903, USA; aax6rw@uvahealth.org (R.A.B.); ccw8sd@uvahealth.org (C.C.W.);; 2 Department of Physical Medicine and Rehabilitation, University of Virginia, Charlottesville, VA 22903, USA; 3 Department of Neurology, Mayo Clinic, Rochester, MN 55905, USA; 4 Division of Gastroenterology, University of Virginia, Charlottesville, VA 22903, USA; mtw3p@uvahealth.org (M.T.W.);; 5 Division of Infectious Diseases and International Health, University of Virginia, Charlottesville, VA 22903, USA

**Keywords:** *Clostridioides difficile* infection, guideline adherence, recurrent *C. difficile*, antimicrobial stewardship, fecal microbiota transplantation

## Abstract

Treatment guidelines for *Clostridioides difficile* infection (CDI) have been published by infectious disease and gastroenterology professional societies; however, adherence in clinical practice remains poorly characterized, particularly for recurrent disease. We conducted a retrospective chart review of 100 patients with CDI (350 episodes: 115 initial, 235 recurrent) referred to a tertiary complicated CDI clinic between 2018 and 2023. Guideline adherence was assessed by comparing treatment with IDSA/SHEA and ACG recommendations, and referring diagnoses were compared with final specialist diagnoses. Guideline adherence was significantly higher in initial compared to recurrent episodes (70.4% vs. 41.3%, *p* < 0.0001). Among guideline non-adherent recurrent episodes, 51.3% used standard antibiotic regimens inappropriate for the recurrence tier. Specialist review reclassified 12.0% of episodes, with colonization increasing from 2.6% to 8.9%. Misdiagnosed colonization cases had a 6.2-fold higher treatment failure rate than confirmed CDI (39.3% vs. 6.3%, *p* < 0.0001). Guideline non-adherence showed a non-significant trend toward treatment failure (10.0% vs. 6.7%, *p* = 0.31). Guideline adherence for recurrent CDI is inadequate in pre-referral settings, and diagnostic misclassification is common. Early specialist involvement may improve both diagnostic accuracy and treatment appropriateness for patients with recurrent CDI.

## 1. Introduction

*Clostridioides difficile* infection (CDI) represents a substantial public health burden within the United States (U.S.) and globally, characterized by significant mortality, high rates of recurrence, and considerable healthcare costs. CDI affects approximately 500,000 individuals in the U.S. annually and causes as many as 30,000 deaths annually [1], being described by the Centers for Disease Control and Prevention as a public health threat that requires “urgent and aggressive action” [2]. The cost of CDI to healthcare systems is also substantial, with direct healthcare costs estimated at 4.8 billion USD annually [3]. Recurrent CDI (rCDI) remains a significant risk as well, with an estimated 10–20% of patients experiencing at least one recurrence following an initial episode, and recurrence rates increasing with each subsequent episode [4]. Some studies have shown that the risk of complications (including sepsis, colectomy, etc.) also increases with each subsequent recurrence, up to 31% per recurrence [5].

rCDI in particular poses a clinical challenge for several reasons. *C. difficile* is known to create spores that may survive traditional antibiotic therapy, remain dormant for some time, and subsequently cause CDI recurrences [6,7]. Gut microbiome disturbances (from antibiotics, previous CDI) that cause increased susceptibility to rCDI generally do not fully resolve by the time appropriate antibiotic treatment has concluded, leading to prolonged susceptibility to further recurrences [8,9,10,11]. Thus, rCDI requires alternate and, likely, more complex treatment regimens compared to initial episodes [12], and professional society guidelines have proposed distinct recommendations for escalating therapy in recurrent cases. Real-world adherence to these recommendations remains poorly characterized.

The Infectious Diseases Society of America and the American College of Gastroenterology publish treatment guidelines for treating initial, recurrent, and multi-recurrent CDI. While initial episodes are treated with a standard course of vancomycin or fidaxomicin, first recurrences are recommended to be treated with a tapered/pulsed vancomycin or fidaxomicin regimen. For patients with multiple recurrences, guidelines recommend consideration of fecal microbiota transplantation (FMT) after failure of antibiotic-based approaches [13,14].

Despite these published evidence-based recommendations, adherence to guidelines for CDI treatment remains suboptimal. Previous research suggests that adherence has historically been low, though recent studies indicate gradual improvement [15,16,17]. However, patterns of adherence, particularly in recurrent CDI, have not been well characterized before patients reach specialized care. Understanding adherence patterns in patients ultimately requiring tertiary care for recurrent CDI may identify opportunities to optimize earlier management and potentially prevent multiple recurrences and their consequences.

This study examined guideline adherence and diagnostic accuracy in 100 patients with initial and recurrent CDI referred to a tertiary care clinic for complicated CDI between 2018 and 2023. We assessed the frequency of guideline-adherent treatment across initial and recurrent episodes, evaluated diagnostic accuracy by comparing referring diagnoses with final specialist diagnoses, and analyzed the relationships between diagnostic agreement, guideline adherence, and treatment outcomes.

## 2. Materials and Methods

### 2.1. Study Design and Setting

This retrospective chart review study was conducted at the University of Virginia Health System, a tertiary academic medical center. The study was approved by the University of Virginia Institutional Review Board. We reviewed medical records of patients evaluated at the Complicated *Clostridioides difficile* Clinic (CCDC) between 2018 and 2023. The CCDC is a specialized clinic that receives referrals from both within the University of Virginia health system and from external providers for management of recurrent and refractory CDI. FMT was considered for patients with multiple CDI recurrences who had failed appropriate antibiotic-based therapy. The decision to recommend FMT was made on a case-by-case basis by the CCDC specialist team, considering recurrence history, prior treatment adequacy, patient comorbidities, and patient preference.

### 2.2. Patient Selection

All patients referred to and evaluated at the CCDC between 2018 and 2023 were eligible for inclusion. We included the first 100 consecutive patients who met the criteria and had sufficient medical record documentation for analysis. For each patient, we collected data on all documented CDI episodes, including those occurring before and after CCDC evaluation.

### 2.3. Data Collection

Medical records were reviewed using the Epic electronic medical record system. When available, records from outside healthcare facilities were accessed through Care Everywhere and incorporated into the analysis. Three medical students performed structured data abstraction using a standardized REDCap database. Data elements collected included patient demographics, comorbidities, details of each CDI episode (date, clinical presentation, diagnostic testing, treatment regimen, and outcomes), timing of CCDC referral, and final CCDC diagnosis.

### 2.4. Variable Definitions

#### 2.4.1. CDI Episodes

Episodes were classified as “initial” if they represented the patient’s first documented CDI episode or if they occurred ≥12 months after a previous episode. Episodes occurring <12 months after a prior episode were classified as “recurrent.” This definition of initial versus recurrent was based on the CCDC treatment guidelines for CDI, with patients presenting with CDI more than 12 months from their most recent CDI episode being treated according to the initial CDI treatment guidelines. Episode dates were determined by the date of positive diagnostic testing or clinical diagnosis as documented in the medical record.

#### 2.4.2. Diagnostic Categories

Each episode was assigned both a referring diagnosis (as documented by the initial treating provider) and a final diagnosis (as determined by CCDC specialist assessment). Diagnostic categories included: (1) confirmed CDI, (2) *Clostridioides difficile* colonization (positive testing without CDI as cause of diarrhea), or (3) non-CDI diarrhea. The final CCDC diagnosis was based on specialist review of clinical presentation, diagnostic testing, response to treatment, and consideration of alternative diagnoses.

#### 2.4.3. Comorbidity Assessment

The Charlson Comorbidity Index (CCI) was calculated for each patient through systematic chart review of documented comorbidities. Age-adjusted CCI scores were calculated according to standard methodology.

#### 2.4.4. Treatment Response

Treatment outcomes were assessed based on documentation at the next healthcare encounter following treatment initiation, which included CCDC follow-up visits, primary care visits, or other clinical encounters where CDI status was addressed. Outcomes were classified as: (1) treatment-responsive (symptoms resolved or improved), (2) treatment-unresponsive (symptoms unchanged, worsened, or resulted in hospitalization or death), or (3) unknown (insufficient documentation to determine outcome). The timing of outcome assessment varied by episode, depending on the documented follow-up patterns in the medical record.

#### 2.4.5. Episode Severity Classification

Episode severity was classified according to ACG and IDSA/SHEA 2021 criteria [13,14]. Non-severe CDI was defined as episodes not meeting criteria for severe disease (WBC < 15,000 cells/mm^3^ and serum creatinine ≤ 1.5 mg/dL). Severe CDI was defined by the presence of WBC ≥ 15,000 cells/mm^3^ or serum creatinine > 1.5 mg/dL. Fulminant CDI was defined as severe CDI with hypotension or shock, ileus, or megacolon, or episodes explicitly documented as fulminant in the clinical note. Episodes with insufficient laboratory or clinical data to classify severity were categorized as unknown.

#### 2.4.6. Diagnostic Testing Classification

Diagnostic testing for each episode was classified based on available documentation. ‘Positive testing’ was defined as a positive result on any CDI-associated assay, including PCR (nucleic acid amplification testing), GDH antigen testing, and/or toxin immunoassay. ‘Negative testing’ was defined as a negative result on all assays performed for that episode. ‘No testing/unknown’ included episodes in which no diagnostic testing was documented. This category encompassed two common scenarios: episodes in which community providers initiated empiric CDI treatment based on clinical presentation in patients with a recent confirmed CDI episode and recurrence of characteristic symptoms, and episodes managed at outside facilities where testing was referenced in clinical documentation, but actual laboratory results were not available for review. 

No single diagnostic algorithm was mandated across all episodes, as patients were diagnosed and initially managed across multiple healthcare facilities prior to CCDC referral. At the University of Virginia Health System, the PCR platform was used between November 2013 and June 2018. Beginning February 2020, UVA Health transitioned from PCR-only testing to multistep PCR with reflex (if PCR+) to toxin enzyme immunoassay (Alere *C. DIFF* QUIK CHEK COMPLETE^®^, TechLab, Inc., Blacksburg, VA, USA). However, the majority of episodes were initially diagnosed at outside facilities where specific diagnostic algorithms and assay manufacturers were not consistently documented in available records. GDH testing detects the presence of *C. difficile* antigen (common to both toxigenic and non-toxigenic strains), while toxin immunoassays detect *C. difficile* toxins A and/or B. The specific toxin immunoassay platforms and targets (toxin A, toxin B, or both) used at referring facilities could not be reliably determined from available documentation.

### 2.5. Guideline Adherence Assessment

Guideline adherence was determined by comparing documented treatment regimens with recommendations from the Infectious Diseases Society of America (IDSA) and the American College of Gastroenterology (ACG) guidelines current at the time of each episode. The following guideline versions were used based on episode year: IDSA 2010, ACG 2013, IDSA/SHEA 2018, ACG 2021, and IDSA 2021 [13,14,18,19,20]. A single investigator performed all assessments of guideline adherence.

Episodes were classified as guideline-adherent if the initial treatment regimen aligned with any recommendation in the applicable guidelines for that episode classification (initial vs. recurrent) and disease severity. Episodes in which the initial regimen was guideline-adherent but subsequently modified due to treatment failure were still classified as adherent, as the initial management followed recommendations. Conversely, episodes where initial treatment was non-adherent but later changed to guideline-adherent therapy were classified as non-adherent based on initial management. For episodes with incomplete dosing or duration information, adherence was classified as “unknown” if available information was insufficient to determine guideline adherence, and as “non-adherent” if available information clearly indicated deviation from guidelines, regardless of missing details.

Treatment regimens were separately categorized as ‘standard’ or ‘non-standard’ based on dosing and duration. ‘Standard’ regimens followed a IDSA/ACG dosing protocol for CDI treatment, including: oral vancomycin 125 mg four times daily for 10–14 days (standard course), vancomycin taper-pulse course (125 mg four times daily tapered to every 2–3 days over 7–15 weeks), fidaxomicin 200 mg twice daily for 10 days (standard course), fidaxomicin extended pulsed course (200 mg twice daily for 5 days followed by 200 mg every other day for days 7–25), or oral metronidazole 500 mg three times daily for 10–14 days. ‘Non-standard’ regimens included those with dosing, frequency, or duration that deviated from any recognized CDI treatment protocol (e.g., abbreviated courses, incorrect dosing, or non-recommended routes of administration).

### 2.6. Statistical Analysis

Descriptive statistics were calculated for patient demographics, episode characteristics, and treatment patterns. Continuous variables were summarized using means and standard deviations or medians and ranges as appropriate. Categorical variables were summarized using frequencies and percentages. Comparisons between groups were performed using Fisher’s exact test or chi-square test for categorical variables and independent t-tests for continuous variables. Statistical significance was defined as *p* < 0.05. No adjustments were made for multiple comparisons. McNemar’s test was used for pairwise comparisons of paired categorical data between referring and final diagnoses, and the McNemar-Bowker test of symmetry was used to assess overall shifts in diagnostic classification. Analyses were performed using GraphPad Prism version 10.5.0 (GraphPad Software, Boston, MA, USA).

## 3. Results

### 3.1. Patient Characteristics

The study cohort comprised 100 patients with recurrent CDI referred to the CCDC between 2018 and 2023 (Table 1). The mean age was 67.3 years (SD 17.8), and 68% were female. The majority of patients were White (92%) and non-Hispanic/Latino (98%). The cohort had a substantial comorbidity burden, with a mean Charlson Comorbidity Index of 4.7 (SD 3.1) and a median of 4.0 [IQR 2.0–7.0]. Each patient experienced a mean of 3.5 CDI episodes (SD 1.8; median 3.0), totaling 350 episodes analyzed: 115 (32.8%) initial and 235 (67.2%) recurrent episodes. Most episodes were non-severe (65.1%), with 15.7% being severe, 2.3% being fulminant, and 16.9% being of unknown severity. Hospitalization occurred in 28.0% of episodes, with no significant difference between initial and recurrent episodes (χ^2^ = 0.247, *p* = 0.619).

The most common referring providers were primary care (46, 46.0%), followed by hospital-based providers (26, 26.0%), gastroenterology (18, 18.0%), other specialties (7, 7.0%), and infectious disease (3, 3.0%).

Diagnostic testing showed positive results in 288 episodes (82.3%), negative results in 13 episodes (3.7%), and no testing documented in 49 episodes (14.0%). FMT was recommended in 57 episodes (16.6%) and completed in 26 patients (45.6%). Breakdowns of diagnostic testing can be found in Table 2.

**Table 2:** Diagnostic Testing Modalities for CDI Episodes. Frequency of diagnostic test types used across all CDI episodes (*N* = 350). Categories are non-exclusive; episodes may be counted in multiple categories if multiple test types were performed. PCR (±toxin assay) includes all episodes with PCR testing regardless of toxin assay use. GDH (±toxin assay) includes all episodes with GDH testing regardless of toxin assay use. Toxin assay includes all episodes with toxin testing. “Testing performed but no specific assay documented” refers to episodes in which a positive or negative CDI test result was referenced in clinical documentation (e.g., in a referring provider’s note or patient history), but the specific assay type (PCR, GDH, toxin) could not be determined because the original laboratory results were from outside facilities and not available for review within our medical record system. “Unknown” refers to episodes with no documented diagnostic testing.

Testing Category	*N* Episodes	% of Episodes
PCR (±toxin assay)	184	52.6%
GDH (±toxin assay)	67	19.1%
Toxin assay	169	48.3%
Testing performed but no specific assay documented	45	12.9%
Unknown	13	3.7%

### 3.2. Overall Guideline Adherence Patterns

**Figure 1:**
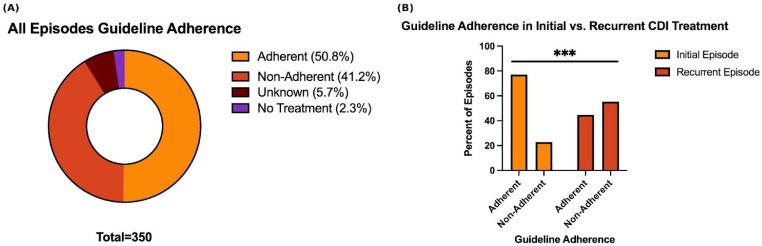
Guideline Adherence Patterns in Initial and Recurrent CDI Episodes. (**A**) Distribution of guideline adherence status among all 350 CDI episodes. (**B**) Comparison of guideline adherence between initial and recurrent CDI episodes shown as percentages of episodes with definitive adherence classification (excluding unknown and no treatment). Initial episodes vs. recurrent episodes (χ^2^ = 30.13, *** *p* < 0.0001).

Among all 350 episodes, 178 (50.8%) received guideline-adherent treatment, 144 (41.2%) received non-adherent treatment, 20 (5.7%) had unknown adherence status, and 8 (2.3%) received no CDI-directed treatment (Figure 1A). When restricting analysis to episodes with definitive adherence classification (excluding unknown and no treatment categories), the overall guideline adherence rate was 55.8% (178/319). These patterns differed substantially between initial and recurrent episodes, as detailed below.

### 3.3. Initial Versus Recurrent Episode Adherence

Guideline adherence differed markedly between initial and recurrent episodes. Among initial episodes, 81 of 115 (70.4%) received guideline-adherent treatment, 24 (20.9%) received non-adherent treatment, 9 (7.8%) had unknown adherence, and 1 (0.9%) received no treatment (Figure S1a). In contrast, among recurrent episodes, only 97 of 235 (41.3%) received guideline-adherent treatment, while 120 (51.1%) received non-adherent treatment, 11 (4.7%) had unknown adherence, and 7 (3.0%) received no treatment (Figure S1b).

When comparing episodes with definitive adherence classifications (excluding no treatment and unknown treatment subgroups), initial episodes demonstrated significantly higher guideline adherence than recurrent episodes (77.1% vs. 44.7%, χ^2^ = 30.13, *p* < 0.0001; Figure 1B). There is a 32.4 percentage-point decrease in adherence for recurrent episodes compared with initial episodes.

### 3.4. Characterization of Non-Adherent Treatment Regimens

Antibiotic regimens were further classified as standard or non-standard based on dosing and duration. Among all episodes, 242 (69.1%) received standard antibiotic regimens (recognized dosing for some indication), 55 (15.7%) received non-standard regimens (dosing or duration not following recommended protocols), and 53 (15.1%) had insufficient documentation to classify (Figure S2a). Notably, all 55 episodes with non-standard regimens were also classified as non-guideline-adherent for the episode type.

To further characterize non-guideline-adherent treatment, we assessed whether these regimens used standard antibiotic protocols (appropriate dosing for a different indication) or non-standard protocols (incorrect dosing or duration). Among the 120 non-adherent recurrent episodes, 61 (51.3%) used standard antibiotic regimens that were inappropriate for the episode classification, 40 (33.6%) used non-standard regimens, and 18 (15.1%) had insufficient documentation to classify (Figure S2b). Approximately half of episodes with a non-adherent treatment regimen involved selecting the wrong treatment tier (e.g., using standard vancomycin when a tapered vancomycin regimen was indicated), while one-third involved both inappropriate regimen selection and non-standard dosing.

### 3.5. Temporal Trends in Guideline Adherence

Guideline adherence rates varied considerably across the study period (Table 3). The uneven distribution of episodes across years reflects the study’s retrospective design: episodes from 1999 to 2017 represent historical CDI events identified through chart review of patients later referred to the CCDC, which was established in 2018. The concentration of episodes in 2020 and 2021 coincides with the early COVID-19 pandemic period, while the lower counts in 2022 and 2023 reflect the tail end of the study enrollment window. Among the years with sufficient episode volume for meaningful comparison (2019 to 2022), adherence rates ranged from 45.8% to 75.0%.

### 3.6. Diagnostic Agreement Analysis

**Figure 2:**
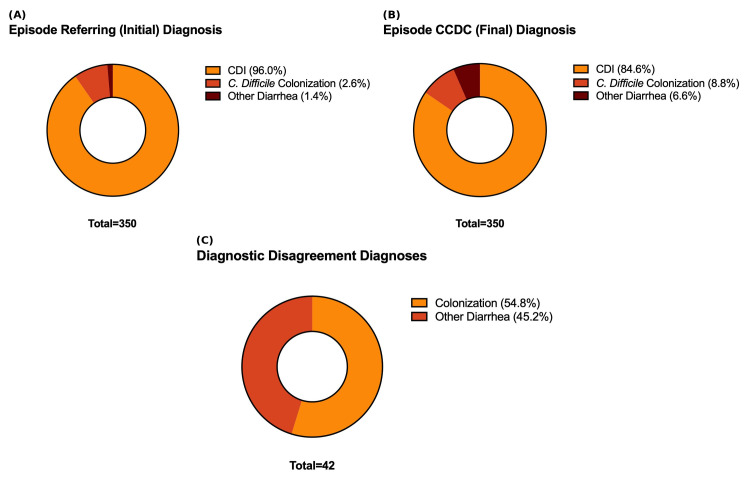
Diagnostic Accuracy: Referring vs. CCDC Specialist Diagnoses and Reclassification of Revised Cases. (**A**) Distribution of referring (initial) community provider diagnoses among all 350 CDI episodes. (**B**) Distribution of diagnoses after CCDC specialist review. (**C**) Final diagnoses among the 42 (12%) reclassified episodes.

**Figure 3:**
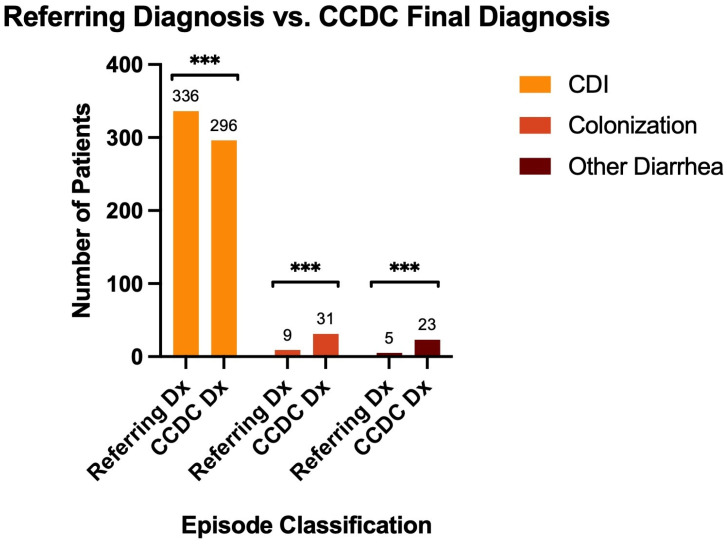
Comparison of Referring Community Provider Diagnoses and Final CCDC Specialist Diagnoses. Grouped bar chart comparing the distribution of referred (initial) clinical diagnoses by community providers to final diagnoses rendered by CCDC specialists across 350 episodes. All pairwise comparisons were statistically significant (*p* < 0.0001, McNemar’s test, denoted ***), with the overall shift in diagnostic distribution confirmed by the McNemar-Bowker test of symmetry (χ^2^ = 36.37, *p* < 0.0001).

Diagnostic accuracy was assessed by comparing referring (initial) clinical diagnoses with final CCDC specialist diagnoses across all 350 episodes. Initial providers diagnosed 336 (96.0%) as confirmed CDI, 9 (2.6%) as colonization, and 5 (1.4%) as non-CDI diarrhea (Figure 2A). Following specialist review, final diagnoses were 296 confirmed CDI (84.6%), 31 colonization (8.9%), and 23 non-CDI diarrhea (6.6%) (Figure 2B). The proportion diagnosed as confirmed CDI thus decreased from 96.0% to 84.6%, while colonization increased 3.4-fold (2.6% to 8.9%) and non-CDI diarrhea increased 4.6-fold (1.4% to 6.6%). Among the 336 episodes initially diagnosed as CDI, 42 (12.0%) were reclassified by specialists: 23 as colonization and 19 as non-CDI diarrhea (Figure 2C). Conversely, 2 episodes initially diagnosed as colonization or non-CDI diarrhea were reclassified as confirmed CDI. Overall, diagnostic disagreement was nearly unidirectional, with reclassification overwhelmingly shifting diagnoses away from CDI (Figure 3).

### 3.7. Treatment Outcomes by Final Diagnosis

**Figure 4:**
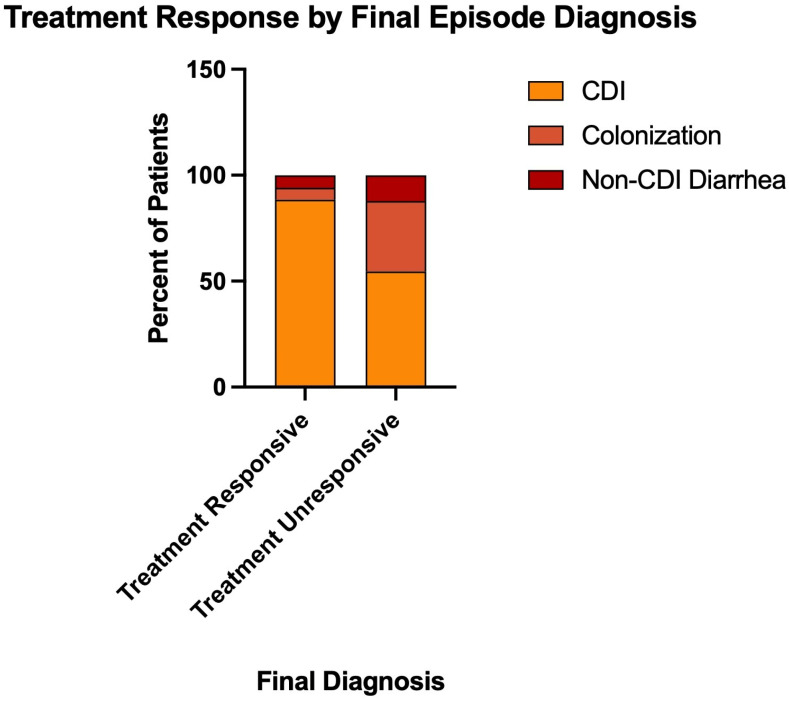
Treatment Response by Final Episode Diagnosis. Treatment outcomes stratified by final CCDC specialist diagnosis among episodes diagnosed as confirmed CDI (285 with known outcomes). Treatment response differed significantly by diagnosis (Fisher’s exact test, *p* < 0.0001), demonstrating that diagnostic accuracy has direct therapeutic consequences.

Treatment response rates differed significantly by final CCDC specialist diagnosis (Fisher’s exact test, *p* < 0.0001; Figure 4). Among 285 episodes diagnosed as confirmed CDI with known treatment outcomes, 267 (93.7%) were treatment-responsive, and 18 (6.3%) were unresponsive. In contrast, among 28 episodes diagnosed as colonization, only 17 (60.7%) responded to treatment, while 11 (39.3%) were unresponsive, representing a 6.2-fold higher treatment failure rate compared to confirmed CDI. Among 22 episodes with non-CDI diarrhea, 18 (81.8%) were responsive, and 4 (18.2%) were unresponsive. These findings demonstrate that episodes misdiagnosed as CDI but ultimately determined to be colonization had substantially worse treatment outcomes, with a treatment failure rate of 39.3% compared to 6.3% for confirmed CDI.

### 3.8. Association Between Diagnostic Agreement and Guideline Adherence

**Figure 5:**
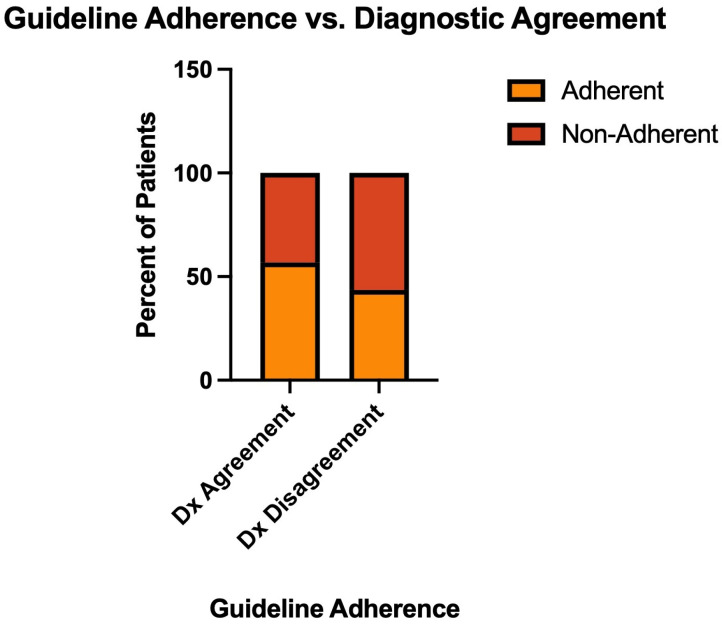
Guideline Adherence vs. Diagnostic Agreement. Comparison of guideline adherence rates between episodes with diagnostic agreement (initial and final diagnoses matched) versus diagnostic disagreement (diagnostic reclassification occurred) among 319 episodes with known adherence status (Fisher’s exact test, *p* = 0.13).

We examined whether episodes with disagreement between initial and final diagnoses (misdiagnoses) were associated with different patterns of guideline adherence. Among 319 episodes with definitive adherence classifications and both diagnoses documented, 280 (87.8%) had agreement in diagnoses, and 39 (12.2%) had disagreement in diagnoses (Figure 5).

Episodes with an agreement in diagnoses showed a guideline adherence rate of 56.8% (159/280), while episodes with disagreement in diagnoses had a numerically lower adherence rate of 43.6% (17/39), representing a 13.2 percentage point difference (Figure 5). However, this difference did not reach statistical significance (Fisher’s exact test, *p* = 0.13), suggesting a trend toward clustering of diagnostic and therapeutic errors but insufficient power to demonstrate this definitively with the available sample size of cases with revised diagnoses.

### 3.9. Treatment Outcomes by Guideline Adherence

**Figure 6:**
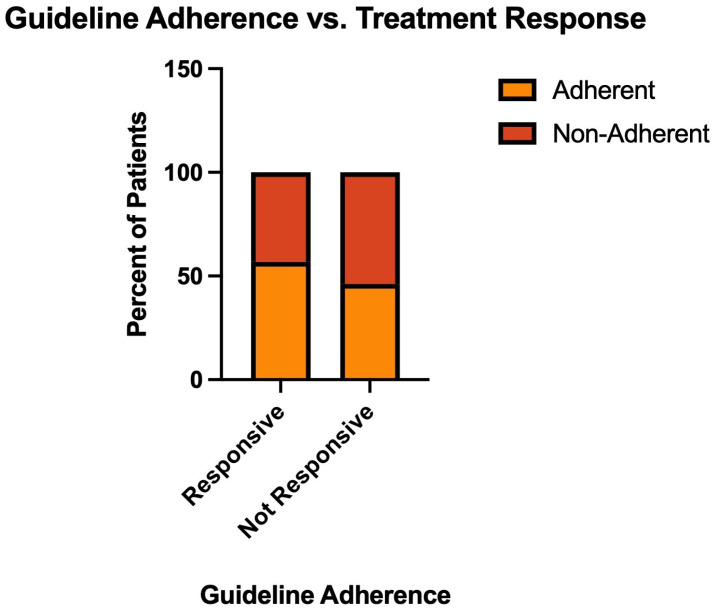
Guideline Adherence vs. Treatment Response. Guideline adherence stratified by treatment outcome. (Fisher’s exact test, *p* = 0.31).

Treatment response was assessed for 318 episodes with known adherence status and documented outcomes. A total of 32 episodes were excluded due to insufficient information to determine either guideline adherence or treatment response. Among guideline-adherent episodes, 166 of 178 (93.3%) were treatment-responsive, and 12 (6.7%) were unresponsive (Figure 6). Among non-adherent episodes, 126 of 140 (90.0%) were treatment-responsive, and 14 (10.0%) were treatment-unresponsive. The difference in response rates was not statistically significant (Fisher’s exact test, *p* = 0.31).

When examining treatment failure rates, non-adherent episodes had a 49% higher relative failure rate compared to adherent episodes (10.0% vs. 6.7%), though this difference did not reach statistical significance (Figure 6). The treatment failure rate in non-adherent episodes (10.0%) approached the failure rate observed in episodes ultimately diagnosed as non-CDI diarrhea (18.2%) and was substantially lower than that seen in colonization (39.3%).

## 4. Discussion

This retrospective analysis of 100 patients with initial and recurrent CDI referred to a tertiary care specialized clinic revealed substantial gaps in guideline adherence, particularly pronounced for recurrent episodes. We additionally identified diagnostic reclassifications, particularly overdiagnosis of colonization as true infection, which is significantly associated with significantly worse treatment outcomes. While guideline non-adherence was associated with a nearly 50% relative increase in treatment failure rates, this difference did not reach statistical significance, likely reflecting survivorship bias and the complex nature of patients requiring tertiary care referral.

### 4.1. Guideline Adherence Patterns in Recurrent CDI

The dramatic decline in adherence to guidelines for recurrent CDI represents a critical quality gap in community practice. Our finding of 41.3% adherence for recurrent episodes is notably lower than the 70.4% observed for initial episodes, suggesting that the complexity of management recommendations for recurrent episodes may impede implementation. Research on rates of guideline adherence in CDI treatment is limited, with prior studies indicating rates as low as 47% in severe cases. More recent data have demonstrated a significant increase in fidaxomicin utilization overall in the last few years [15,16]. Our study suggests that while adherence for initial CDI may be improving nationally, management of recurrent disease, where treatment escalation is most critical, remains inadequate in pre-referral settings.

Notably, half of the non-adherent recurrent episodes used standard antibiotic regimens that were inappropriate for the episode tier; for example, using standard-course vancomycin when tapered vancomycin was indicated. This pattern suggests that the primary barrier is not inappropriate antibiotic selection per se, but rather failure to escalate therapy according to recurrence status. This discrepancy may reflect limited familiarity with tapered regimens, concerns about treatment duration and complexity, or lack of recognition that standard regimens are insufficient for recurrent disease.

### 4.2. Diagnostic Accuracy and Clinical Consequences

The challenge of distinguishing CDI from colonization is well-recognized in the literature. The widespread adoption of nucleic acid amplification tests (NAATs) since 2009 has dramatically improved sensitivity but at the cost of specificity for clinically significant disease [21]. NAAT-based testing detects toxin genes but cannot distinguish between active toxin production and asymptomatic carriage/colonization, leading to potential overdiagnosis when applied to patients with diarrhea from alternative causes [21,22]. Available literature reports that approximately 25% of patients referred for recurrent CDI ultimately receive alternative diagnoses, with post-infectious irritable bowel syndrome and inflammatory bowel disease being common alternative etiologies [23].

Our data contribute to growing evidence that diagnostic stewardship (i.e., ensuring appropriate patient selection for testing and proper interpretation of results) is essential to CDI management [24]. The 12.0% reclassification rate in our cohort likely underestimates the true prevalence of misdiagnosis, as our study was limited to patients who eventually reached specialist evaluation. Many patients with misdiagnosed CDI may never be referred for specialized assessment, continuing to receive inappropriate treatment indefinitely.

The consequent treatment failure in misdiagnosed colonization cases underscores the harm caused by diagnostic inaccuracy. Patients with colonization receiving anti-CDI antibiotics experience unnecessary drug exposure, further microbiome disruption, healthcare costs, and delayed diagnosis of their actual condition. Moreover, colonization can persist after CDI treatment completion and remain detectable by NAAT testing, likely contributing to reported CDI recurrence rates in epidemiologic studies and potentially overestimating the true burden of recurrent disease [25].

### 4.3. Treatment Outcomes and Guideline Adherence: A Complex Relationship

Contrary to our initial hypothesis, guideline non-adherence was associated with only a modest and non-significant increase in treatment failure rates. This finding likely reflects several study design limitations discussed below rather than indicating that guideline adherence is unimportant. The nature of non-adherence in our cohort may partly explain the modest outcome differences. Notably, about half of non-adherent recurrent episodes used standard antibiotic regimens at inappropriate treatment tiers rather than truly aberrant therapies. These regimens, while insufficient for recurrent disease, may provide temporary symptom control even if they fail to prevent future recurrences. This “wrong tier, right drug” pattern likely has less dramatic immediate consequences than the use of completely inappropriate agents. The intermediate failure rate in non-adherent episodes (10.0%) falls between that of confirmed CDI (6.3%) and colonization (39.3%), consistent with a mixed population of suboptimally treated infections and inappropriately treated colonization.

### 4.4. FMT Utilization and Advanced Therapy Implementation

Despite guideline recommendations for FMT after failure of appropriate antibiotic-based therapy for recurrent CDI, FMT was recommended in only 57 episodes (24.3% of recurrent episodes) and completed in 26 patients (45.6% of recommended cases). The relatively low recommendation rate reflects the CCDC’s approach of first optimizing medical therapy in patients referred with prior non-adherent treatment, particularly through extended vancomycin taper-pulse regimens, which have demonstrated efficacy of up to 80% [26]. Among patients for whom FMT was recommended but not completed (31 cases), barriers included patient reluctance, logistical challenges such as scheduling and bowel preparation requirements, cost, and safety concerns. The FDA approval of standardized microbiome-based therapeutics (Rebyota, Vowst) in 2022–2023 may improve access and uptake going forward [7,27,28,29,30,31,32].

### 4.5. Limitations

Several important limitations warrant consideration. Firstly, the single-center, retrospective design limits generalizability and introduces potential selection bias. Our population represents patients successfully referred to and evaluated by a tertiary care clinic, systematically excluding patients who died, underwent colectomy, or were managed entirely in other systems. This survivorship bias likely results in an underestimation of the consequences of non-adherent therapy.

Secondly, the guideline adherence classification relied on a retrospective chart review and the research team’s assessment. While we used published IDSA/SHEA and ACG guidelines as our standard, clinical judgment was required, particularly for episodes with incomplete documentation [13,14,28,29,30]. Further, diagnostic reclassification by the specialist team, while reflecting expert opinion, does not represent a perfect gold standard. Beyond this, the heterogeneity of diagnostic testing across referring facilities precluded standardized assessment of diagnostic algorithms, specific assay platforms, and toxin targets.

Thirdly, treatment outcome assessment was based on variable follow-up intervals and potentially incomplete documentation. The distinction between “resolved” and “improved” symptoms may be subjective, and long-term outcomes, including subsequent recurrence prevention, were not systematically captured. Additionally, outcome timing varied, with some assessments occurring within days of the initial treatment, whereas others occurred weeks or months later.

## 5. Conclusions

This study documents substantial, clinically important gaps in CDI guideline adherence, particularly pronounced in recurrent episodes, where appropriate therapy escalation is most critical. While we could not demonstrate a statistically significant relationship between guideline adherence and immediate treatment outcomes, likely due to survivorship bias and study design limitations, the observed patterns suggest that inappropriate management contributes to the cycle of recurrence necessitating tertiary care. Our findings provide a foundation for targeted quality improvement initiatives and highlight the ongoing gap between guideline recommendations and real-world practice.

## Figures and Tables

**Table 1:** Patient and Episode Characteristics. Demographic and clinical characteristics of 100 patients referred to the Complicated CDI Clinic between 2018 and 2023. Data are presented as raw numbers and percentages for categorical variables and mean (standard deviation) or median [interquartile range] for continuous variables.

Characteristic	Value
Number of patients	100
Mean age, years (SD)	67.3 (17.8)
Sex	
Female	68.00%
Male	32.00%
Race	
White	92.00%
Black	6.00%
Other	2.00%
Ethnicity	
Non-Hispanic/Latino	98.00%
Hispanic/Latino	2.00%
Charlson Comorbidity Index	Mean 4.7 (SD 3.1); Median 4.0 [IQR 2.0–7.0]
Number of CDI episodes per patient	Mean 3.5 (SD 1.8); Median 3.0
Initial CDI episodes	115 (32.9%)
Recurrent CDI episodes	235 (67.1%)
Hospitalization during episodes	98 (28.0%)
Episode severity	
Non-severe	228 (65.1%)
Severe	55 (15.7%)
Fulminant	8 (2.3%)
Unknown	59 (16.9%)
Use of FMT	26 episodes
Diagnostic testing	
Positive Testing	288 Episodes (86%)
Negative Testing	13 episodes (3.7%)
No testing / Unknown	49 episodes (14.0%)
Guideline-adherent treatment	
Initial episodes	81 (70.4%)
Recurrent episodes	95 (40.4%)

**Table 3:** Temporal Trends in Guideline Adherence by Year. Annual distribution of CDI episodes and guideline adherence rates from 1999 to 2023. Episodes are categorized by guideline adherence status: guideline-adherent, non-adherent, no treatment, or unknown adherence. Adherence percentage calculated as the proportion of total episodes per year that received guideline-adherent therapy.

Year	Total Episodes (n)	Guideline-Adherent	Guideline Non-Adherent	No Treatment	Unknown	Adherence (%)
1999	1	0	0	0	1	0.0%
2013	1	1	0	0	0	100.0%
2015	2	0	0	0	2	0.0%
2017	14	5	8	0	1	35.7%
2018	4	2	2	0	0	50.0%
2019	36	27	7	0	2	75.0%
2020	142	65	62	4	11	45.8%
2021	89	45	38	2	4	50.6%
2022	56	26	26	2	2	46.4%
2023	4	4	0	0	0	100.0%

## Data Availability

The data presented in this study are not publicly available due to patient privacy restrictions under HIPAA and the terms of the IRB-approved protocol (HSR23045). Requests for de-identified, aggregate data may be directed to the corresponding author.

## Supplementary Materials

The following supporting information can be downloaded at https://www.mdpi.com/article/10.3390/microorganisms14040911/s1: Figure S1a: Guideline Adherence Distribution in Initial Episodes; Figure S1b: Guideline Adherence Distribution in Recurrent Episodes; Figure S2a: Standard vs. Non-Standard Antibiotic Regimens Across All Episodes; Figure S2b: Antibiotic Classification Among Non-Adherent Recurrent Episodes; Table S1: Antibiotic Treatment Categories for CDI Episodes (*N* = 350).

## Abbreviations

The following abbreviations are used in this manuscript:

CDI	*Clostridioides difficile* infection
rCDI	Recurrent *Clostridioides difficile* infection
CCDC	Complicated *C. difficile* Clinic
