# Supplementary material for: Treating Initial and Recurrent C. difficile: A Retrospective Analysis of 100 Referred Patients
Source: Microorganisms. 2026 Apr 17;14(4):911. doi: 10.3390/microorganisms14040911 (PMC13118397; doi:10.3390/microorganisms14040911)
Supplement: Supplementary file 1 [file microorganisms-14-00911-s001.zip › Table S1.pdf]

Table S1. Antibiotic Treatment Categories for CDI Episodes (N=350). Treatment regimens were classified by antibiotic type (vancomycin, fidaxomicin, metronidazole), dosing pattern (standard short course, standard taper-pulse or extended pulsed course, and non-standard dosing), and other/unknown categories. Categories are non-exclusive; episodes involving multiple antibiotics may appear in more than one category. Percentages are calculated from all 350 episodes.

Treatment Category	N Episodes	%
**Vancomycin**		
Standard short course (125 mg QID x 10–14 days)	144	41.1
Standard taper-pulse course (125 mg QID tapered weekly to every 3 days, 7–15 weeks)	92	26.3
Non-standard dosing	68	19.4
**Fidaxomicin**		
Standard short course (200 mg BID x 10 days)	31	8.9
Standard extended pulsed course (200 mg BID x 5 days, then 200 mg QOD for days 7–25)	2	0.6
Non-standard dosing	11	3.1
**Metronidazole**		
Standard short course (500 mg TID x 10–14 days)	18	5.1
Non-standard dosing	23	6.6
Other*	26	7.4
Unknown†	8	2.3

** "Other" refers to non-standard combinations of multiple CDI and/or non-CDI antibiotics.*

† "Unknown" refers to episodes where CDI diagnosis was documented but specific treatment information was unavailable, typically due to incomplete records from outside health systems.

Categories are non-exclusive; episodes involving multiple antibiotics may appear in more than one category. Percentages are calculated from all 350 episodes.
