# Supplementary figures and images for: Treating Initial and Recurrent C. difficile: A Retrospective Analysis of 100 Referred Patients
Source: Microorganisms. 2026 Apr 17;14(4):911. doi: 10.3390/microorganisms14040911 (PMC13118397; doi:10.3390/microorganisms14040911)

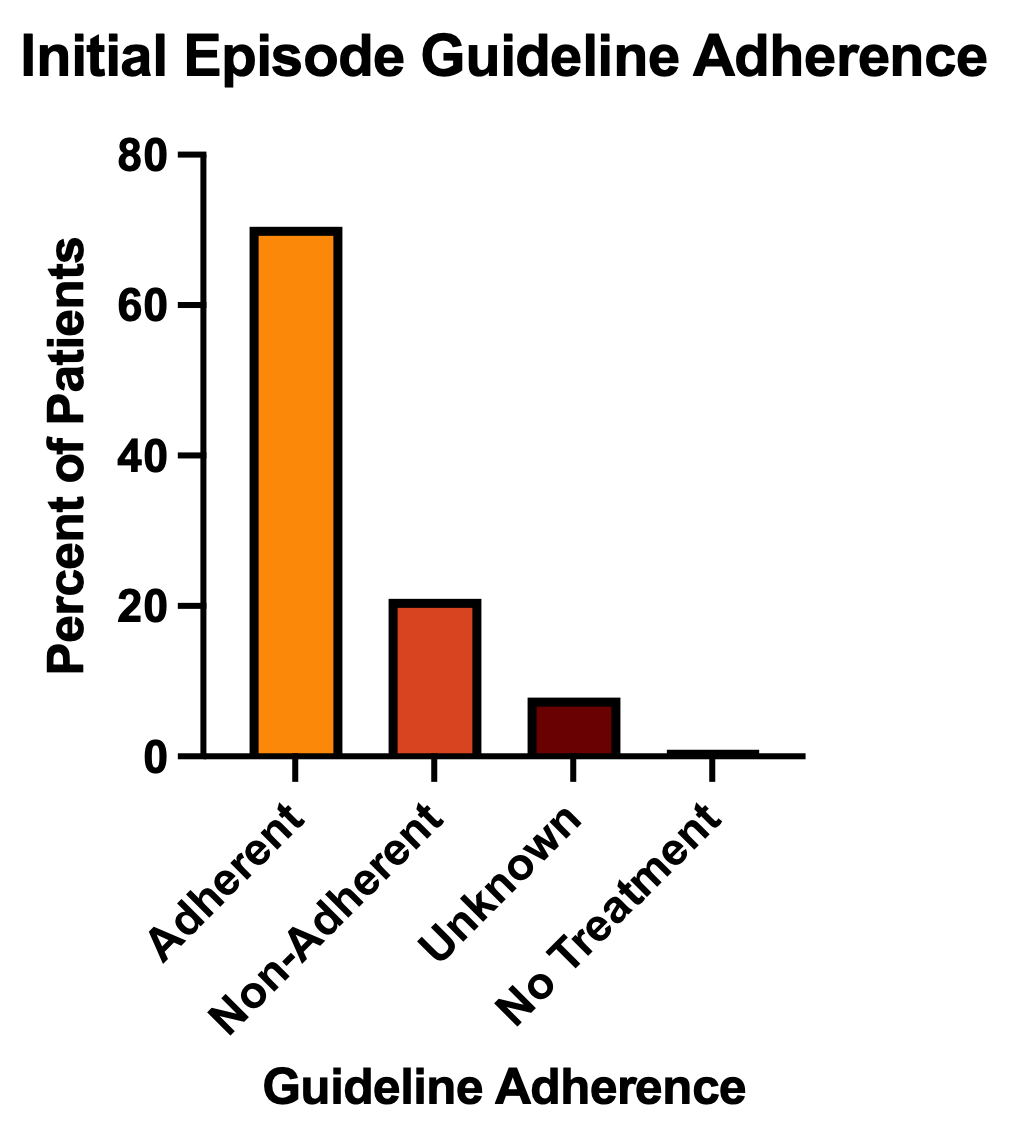

Supplement: Supplementary file 1 [file microorganisms-14-00911-s001.zip › Figure S1a.png]

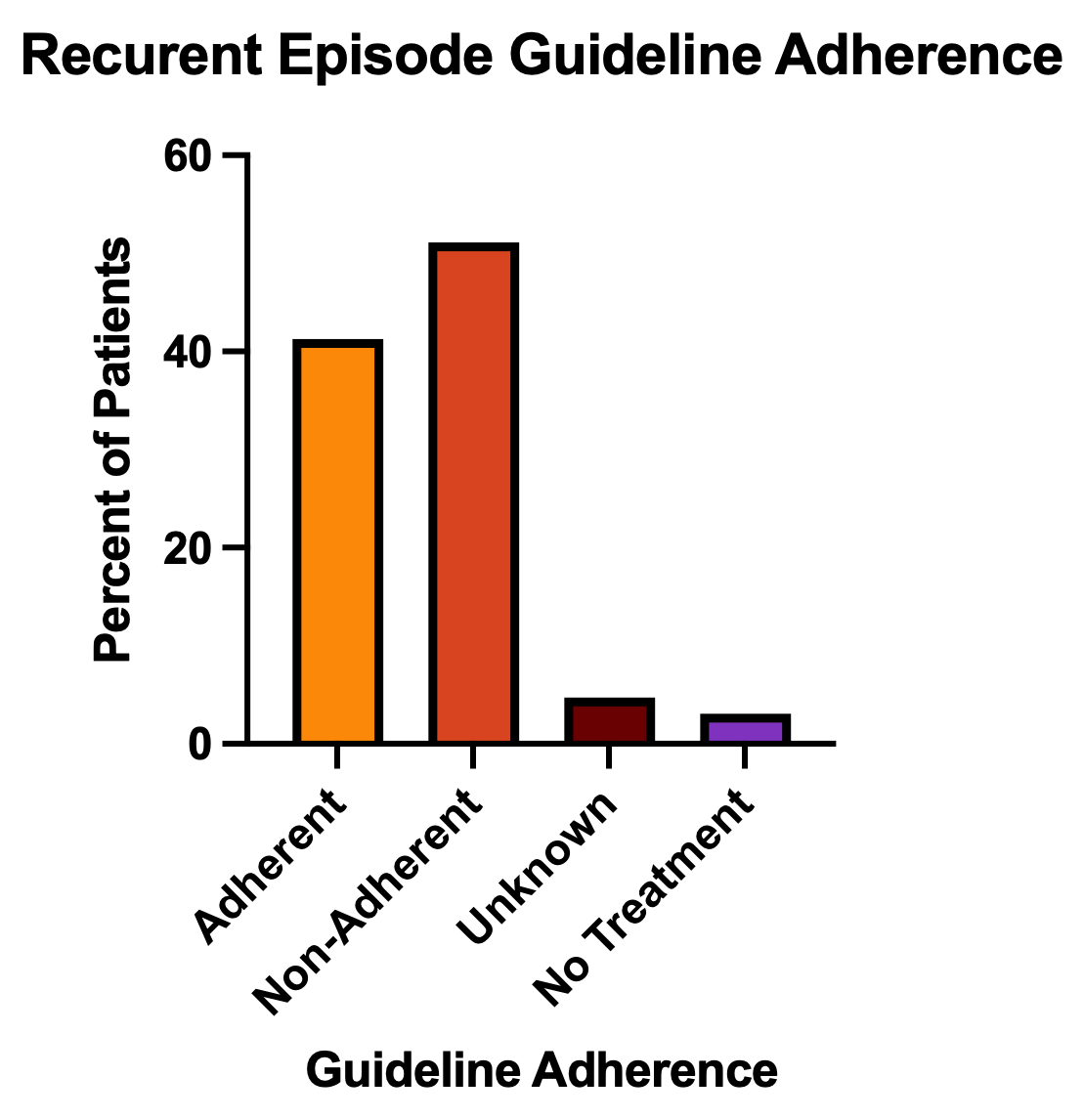

Supplement: Supplementary file 1 [file microorganisms-14-00911-s001.zip › Figure S1b.png]

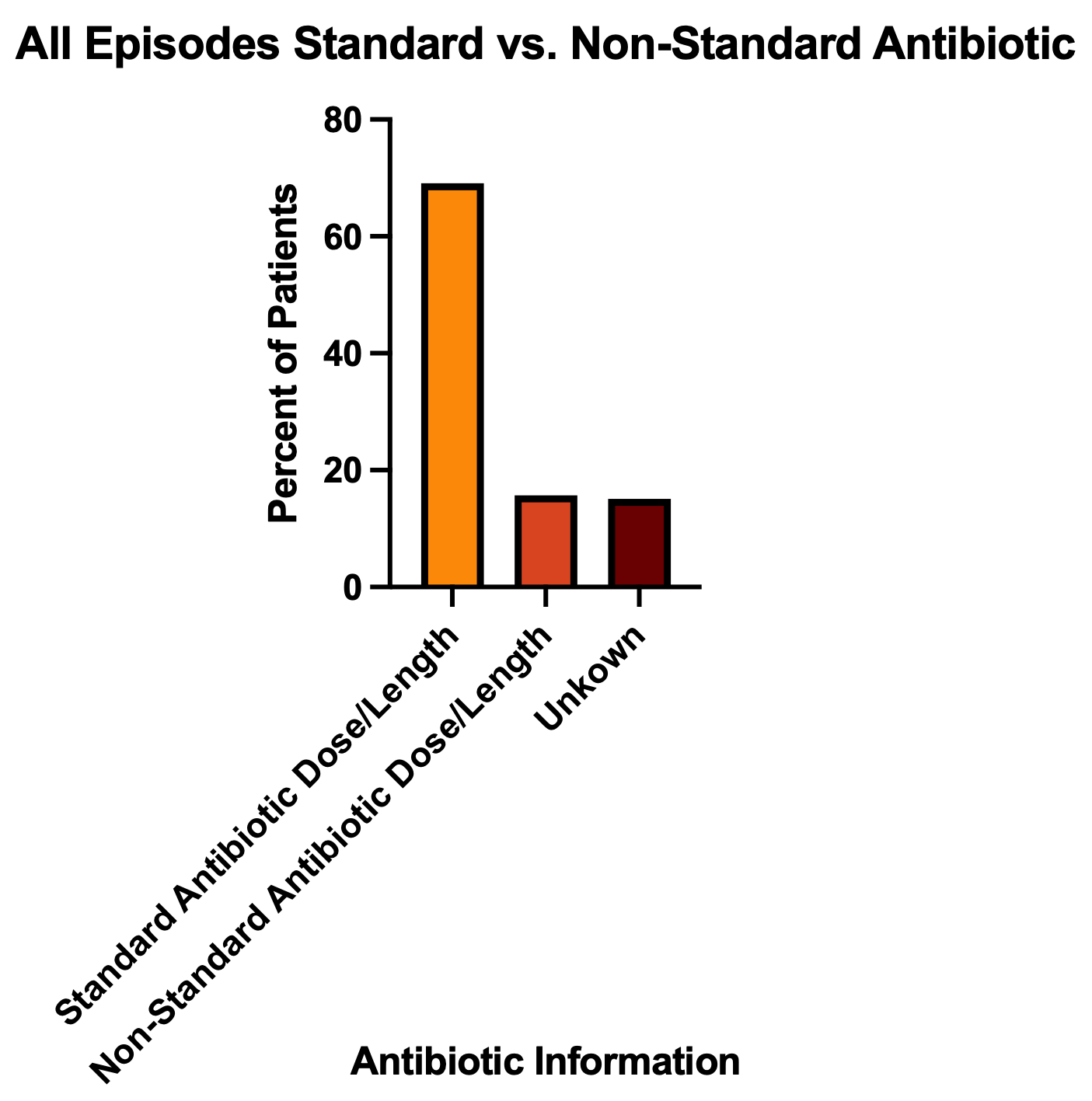

Supplement: Supplementary file 1 [file microorganisms-14-00911-s001.zip › Figure S2a.png]

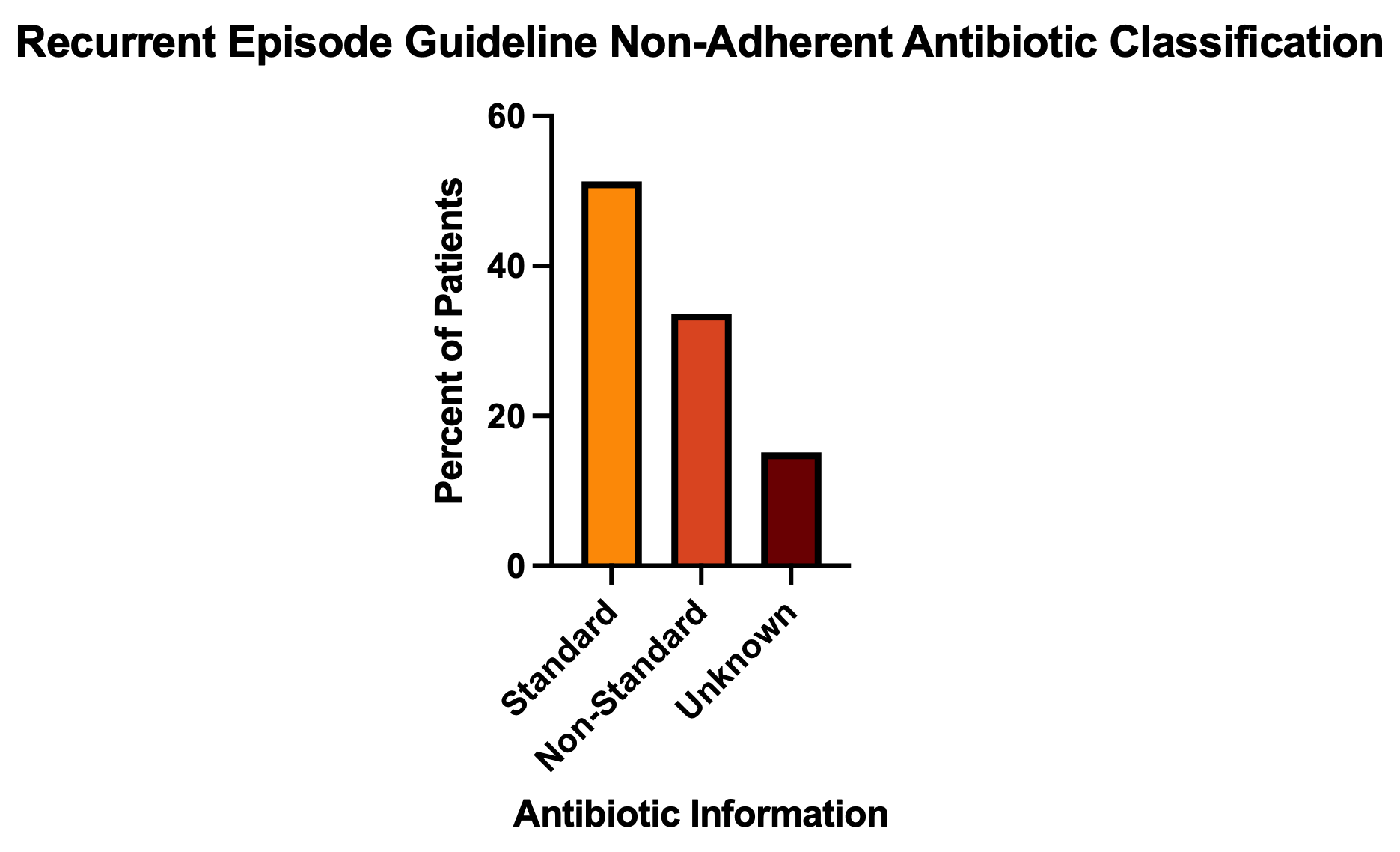

Supplement: Supplementary file 1 [file microorganisms-14-00911-s001.zip › Figure S2b.png]
